# Proposition for Determining the Residual Strength of Fiber-Reinforced Cement Composite

**DOI:** 10.3390/ma15217546

**Published:** 2022-10-27

**Authors:** Wiesława Głodkowska, Joanna Laskowska-Bury

**Affiliations:** Faculty of Civil Engineering, Environmental and Geodetic Sciences, Koszalin University of Technology, Sniadeckich 2, 75-453 Koszalin, Poland

**Keywords:** fiber-reinforced composite, steel fibers, bending, beams, plates, residual strength, test methods

## Abstract

Designing bending elements made of fiber composites requires knowledge of the residual strengths. Residual strengths determined according to PN-EN 14651, regardless of the type of matrix and the fibers used, are characterized by a very-high coefficient of variation, about 30%. The variability of this feature is so large that the normal distribution adopted in statistical analyses, which is consistent for compressive strength or tensile strength, may, in the case of residual strengths, result in a significant overdesign of the elements. Therefore, the article proposes a novel method of determining the residual strength with the use of centrally bent square plates simply supported at the perimeter. The coefficient of variation of this characteristic in the case of plate testing is about 8%.

## 1. Introduction

Due to advantages such as general availability and relatively low production costs, concrete is one of the most commonly used building materials. Unfortunately, it is a brittle material, and its destruction occurs at low deformation values. Due to it slow tensile strength and susceptibility to crack propagation, concrete today, in some cases, is becoming a material with insufficient physical and mechanical properties.

The brittle nature of concrete is a problem for structural designers because the ability of a structure to undergo high plastic deformation before failure can be critical. Therefore, research is constantly being carried out to find solutions to improve the properties of concrete. A way to improve the properties is to modify the concrete mix by adding fiber reinforcement. The very thorough and vast overview of different fibers was reported by Mohajerani et al. [1]. They presented properties of fibers and their applications in construction materials and referred to waste and natural fibers, which are important to the sustainable development point of view.

Composites with the addition of fiber reinforcement have been known for a long time, and sometimes they are an alternative to ordinary concrete and characteristic of better properties. Compared to regular concrete, fiber-reinforced concrete shows higher resistance to dynamic loads [2,3] and high temperatures [4,5], and it is characterized by greater durability [6], resistance to abrasion [7], and less shrinkage [8,9]. However, the addition of fibers has the greatest influence on the tensile strength, which has been discussed, among others, in the works [2,10,11,12,13,14,15]. Added fibers transform brittle concrete into a quasi-plastic material, which is characterized by the possibility of the redistribution of tensile stresses after cracking to near fibers. Thus, after reaching destructive stresses, it does not suddenly break but retains the load-bearing capacity, which is undoubtedly the most important feature determining the suitability of this material in structures. This property enables the use of fiber-reinforced composites for the production of various structural elements, such as prefabricated thin-walled elements, slabs [8,9,16] and foundations [17], as well as industrial pavements [2,18], road and airport pavements [3], and prefabricated and monolithic tunnel linings [19,20,21,22], for which the control of fracture processes is particularly important.

Regardless of the type of fibers or the matrix, in order for the design process to effectively use the ability of fiber-reinforced concrete to transfer load-destructive tension after post-cracking, it is necessary to understand the behavior of the post-cracking elements under tensile stress, which is defined by the residual strengths [23,24,25,26,27].

Many methods have been developed to determine the fracture toughness of fiber-reinforced materials. These include the uniaxial tension test method proposed by RILEM (RILEM TC-162-TDF [28]), the wedge splitting test [29], tests with the use of bent beams (recommendations PN-EN 14651 [30], Model Code 2010 [31], ASTM C 1018 [32], regulations of the Japanese Concrete Institute (JCI-SF4 [33])), and standards allowing the use of plate-shaped samples, for example, ASTM C 1550 [34] and PN-EN 14488-5 [35].

The choice of the method to determine the flexural tensile strength of the fiber composite depends on the adopted calculation method based on specific properties. The most common method for analyzing the behavior of fiber-reinforced concrete after cracking under tensile conditions is the recommendations of RILEM TC 162-TDF [36], which are included in the fib Model Code 2010 [31]. The presented procedures for calculating fiber-reinforced elements are based on the residual strengths determined according to PN-EN 14651 [30].

Unfortunately, based on the experience of the authors presented in the monograph [37] and the analysis of the results of other researchers [38,39,40,41,42,43], it can be observed that the results of residual strength, regardless of the type of matrix and the type of the fibers used, are characterized by a large coefficient of variation. This result is attributed to the uneven distribution of fibers inside the concrete mix, especially in place of the notch of the samples tested for bending [42,44].

For example, N. Buratti et al. [42], while testing the properties of concrete reinforced with steel and macrosynthetic fibers, they obtained statistical variability of the residual strength results at the level of 15–20%. Soetens T. and Matthys, in their study [45], proposed an alternative constitutive relation for those presented in the Model Code [31]. The authors developed a model that considers the intermediate behavior between the reinforcement and the weakening of the fiber-concrete post-cracking. The described model is available in two variants: (1) a trilinear constitutive model, derived by inverse analysis, and (2) an analytical model based on pulling the fiber from the matrix. The model parameters (1) were defined as a function of the residual strengths f_R.1_ and f_R.3_ determined according to [30], whose coefficients of variation were characterized by high variability and ranged from 10 to 33%. M. Pająk [46] researched the effect of fibers from tire recycling on the behavior of concrete. The results showed that the fibers had little effect on the compressive strength, while clearly influencing the bending behavior of the concrete. The residual strengths increased in proportion to the number of fibers; however, with the increase in the fiber content, the scatter in the test results also increased. Z. Zamanzadeh et al. [41], in their study, drew attention to the fact that the tendency of the high variability in the results of residual strength may be even more visible for samples made with the use of recycled steel fibers. This is mainly due to their greater geometrical variability and irregularity in relation to industrially produced fibers. Shan He and En-Hua Yang [47] found that the variability in the residual strengths was so large that the normal distribution adopted in statistical studies, which is consistent for compressive strength or tensile strength, in the case of residual strengths, may result in more than 30% overdesign. This leads to higher material costs and higher production risks.

Large values of the coefficients of variation, which characterize the results of the research on the residual strengths of fine-aggregate fiber composite [48,49] and have been reported in the works of other researchers, prompted the authors to conduct more extensive research. We tested 30 beams with dimensions of 150 × 150 × 700 mm. The research was extended by the analysis of centrally bending square plates simply supported at the perimeter. A total of six plates were tested. It was noticed that the methodology of testing plates (PN-EN 14488-5 [35]) and beams (PN-EN 14651 [30]) and the shapes of the load–deflection graphs (F-δ) were similar. It was assumed that the study of the energy absorption capacity can also be used to determine the residual strengths. This test had a much lower coefficient of variation than the beam test. On this basis, an innovative, more precise method of determining the residual strengths was developed compared to that proposed in PN-EN 14651 [30].

The novel method of testing the residual strength proposed in the article using centrally bent square plates simply supported at the perimeter is aimed at more realistic modeling of biaxial bending than in the case of beams. The obtained residual strength values are characterized by a much lower coefficient of variation (about 8%), and thus this feature is determined with greater reliability. This is because the residual strengths are the basic and most important feature in the design of bending fiber-concrete sections.

## 2. Test Materials

**Figure 1:**
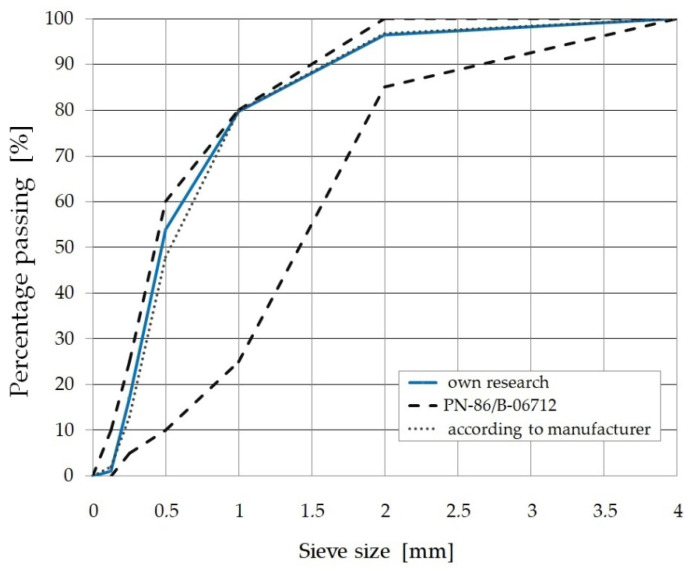
Grading curve of the aggregate used in the research program.

The test elements were made of fine aggregate cement composite with the addition of steel fibers. The fine-aggregate fiber composite is a novel construction material patented No. 239641, “Fine-aggregate mineral composite reinforced with scattered fibres”, Koszalin University, in which the aggregate used is post-production waste. In the analyzed composite, the aggregate was sand with granulation up to 4 mm, which is the waste of aggregate mines located in northern Poland (the Pomerania region) [50,51]. In this area, a significant part of the output is subjected to the process of hydroclassification. It is the technology for obtaining coarse aggregate by washing it out from the deposits. As a result of these actions, 80% sand and only 20% coarse aggregate is obtained. The aggregate used, although it is treated as waste, as shown by the analysis of the results of the tests carried out, meets the requirements for mineral aggregates recommended for the production of ordinary concrete [52,53,54]. The aggregate used is characterized by continuity of the grading curve (Figure 1) and high uniformity of grain size. The content of mineral dust in the aggregate is less than 3%, which allows it to be classified into the f_3_ category based on the PN-EN 12620 standard [55].

The elements for the tests were used as described above: sand (1570 kg/m^3^), Portland cement CEM II/A-V 42.5R (420 kg/m^3^), silica dust (21 kg/m^3^), superplasticizer (16.8 kg/m^3^), and tap water (160 kg/m^3^).Fast-hardening Portland cement containing fly ash was used. Research has shown that fly ash has a positive effect on the properties of concrete mixtures [56,57].

The fiber reinforcement consisted of steel fibers with a hooked shape in the amount of 1.2% in relation to the volume composite (94 kg/m^3^). The aspect ratio of fibers was λ = l/d = 62.5 (l = 50 mm, d = 0.8 mm). The fibers in the mixture were distributed randomly. The fiber content was determined based on the results of previous studies of composites with a fiber content of 0–2.5% [51]. The analyses proved that with the fiber content to be in the amount of 1.2% in relation to the volume composite, the tested fiber composite showed the best mechanical and physical properties. The technical characteristics of the steel fibers used in the studies are discussed in detail in the article [50]. For fine-aggregate composite the ratio was assumed at *w*/*c* = 0.38. The consistency of the fiber composite mixture was determined by the Vebe method according to PN-EN 12350-3 [58] and was V2.

The fiber composite mixture was made under laboratory conditions, in a counterrotating concrete mixer, maintaining the assumed sequence of dosing the components and the specified mixing time:

- aggregate + cement + silica dust—2 min.
- aggregate + cement + silica dust + water and plasticizer—4 min.
- aggregate + cement + silica dust + water and plasticizer + steel fibers—4 min.

The mixture was placed into the molds in two layers, compacting each of them for 30 s using a table vibrator with a frequency of 50 Hz.

The steel fibers were added by spreading them gradually while mixing in order to avoid their concentration. The test elements, after demolding, were stored for 27 days at the temperature of 20 ± 2 °C and arelative air humidity of 100%. Then, until loading (30 days after molding), it was stored under the conditions of 20 ± 2 °C and 50 ± 2%.

The authors have been conducting research on the use of waste sands for the production offine-aggregate fiber composites of structural importance for many years. In the studies [37,52,59,60], the conditions for making samples, their care, and the mechanical–physical properties of fine-aggregate fiber composites have been widely discussed.

## 3. Methodology of Research and Test Elements

The assumed test program included testing the residual strength and the limit of proportionality of the fiber composite with the use of beam elements with dimensions of 150 × 150 × 700 mm and testing the energy absorption capacity of the fiber composite panels with dimensions of 100 × 600 × 600 mm. The necessary minimum number of samples to determine the statistical mean value of the examined feature was determined on the basis of statistical analysis using the Student’s *t*-distribution, with a tolerance of 10% and a significance level of 0.05.

The aim of the experimental research and theoretical analyses was to develop a method of testing the strength of residual fiber composites that would allow for the lowest possible variability of this property. As shown in Section 1, this feature is a basic property in the design of fiber concrete elements, and the residual strength test is burdened with a large coefficient of variation.

### 3.1. Beam Elements

**Figure 2:**
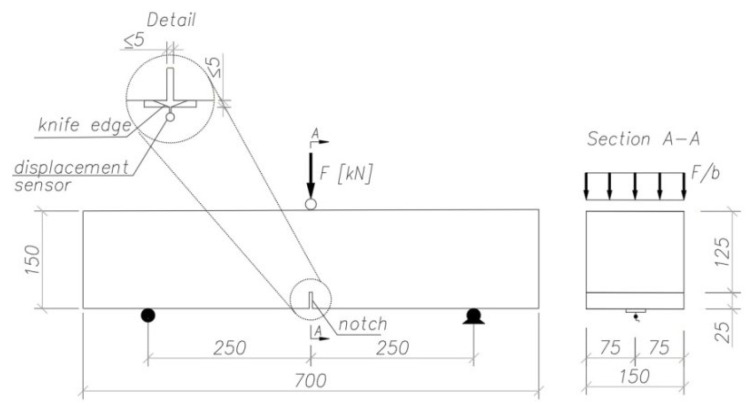
Scheme of the beam load during the testing of the residual strength of the fiber composite [30].

The beam elements were tested according to PN-EN 14651 [30]. The relation of “load–CMOD” determined for the three-point flexural tensile test according to [30] serves to define the residual strengths f_R.1_, f_R.2_, f_R.3_, and f_R.4_. Prior to testing, the samples were incised in the central part to a depth of 25 mm and a width of ca. 3 mm. The test elements were placed on articulated supports with 500 mm spacing. The beams were loaded in half of the span in the use of the scheme recommended by [30] (Figure 2).

The beam load was applied in a continuous manner with a variable rate. The increase in the beam load speed was determined depending on the CMOD crack width. Due to the possibilities of the equipment, the test was performed by applying a load as a function of displacement, while the tested value was the width of the CMOD crack. According to [30], the measured value of the displacement can be both the deflection (δ) and the CMOD. The speed of load increase was controlled by measuring the deflection (δ). This speed was determined in preliminary tests with the standard dependence of CMOD-δ [30]. In the first phase, for a CMOD width of 0.1 mm, the load was applied at a displacement rate of 0.5 mm/min. After exceeding this value, the displacement rate was changed to 0.2 mm/min. The test was conducted until the CMOD was reported to be above 3.5 mm. The deflection and crack width were recorded using the SAD 256 data acquisition system with a set of electrofusion sensors (two sensors with articulated ends and two with invariable ends) and a sensor to measure the deflection. The precision of the sensors was 1 mV/V. The limit deflection value of the beams was determined according to PN-EN 14651 [30] so that all CMOD values could be achieved. Then, the residual strengths (f_R.j_) were determined for the respective CMOD, j, where j = 1, 2, 3, and 4. For the strengths f_R.1_, f_R.2_, f_R.3_, and f_R.4_, the mean tensile stress values in the cross-section for the given CMOD widths were 0.5, 1.5, 2.5, and 3.5 mm, respectively. The values of the residual strengths of the fine-grained fiber composite were determined according to the formula [30]:

f_R.j_ = (3 × F_j_ × l)/(2 × b × hsp^2^) (1)

where h_sp_ is the distance between the notch tip and the top of the specimen; F_j_ is the load corresponding to CMOD_j_; l is the span of the beam; and b is the width of the specimen.

**Figure 3:**
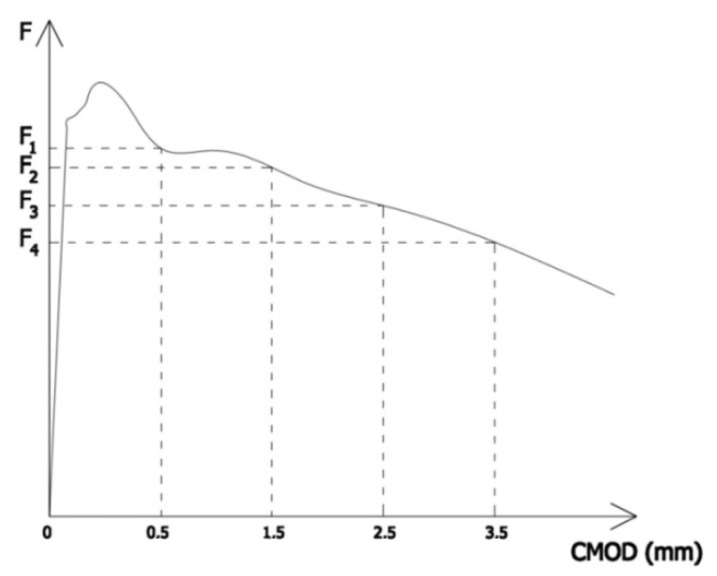
Graphic interpretation of determining the values F_1_ to F_4_ [31].

The graphic interpretation of determining the values of F_1_ to F_4_ is shown in Figure 3 [31].

**Figure 4:**
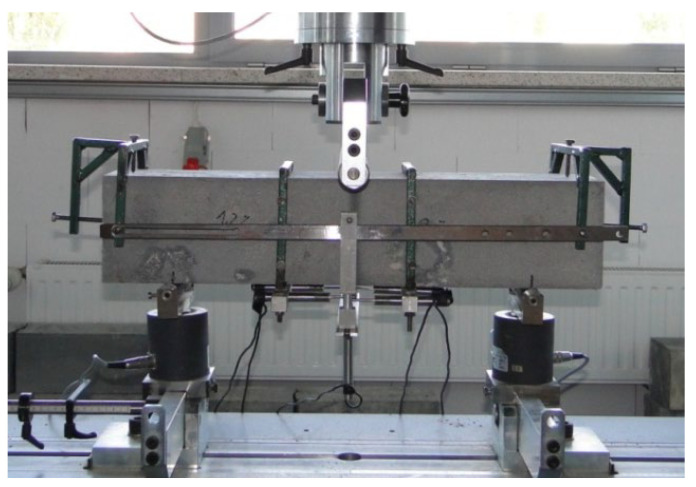
A sample on the fiber composite residual strength test stand.

An important parameter that allows for classifying a fiber composite is the shape of the graph of the load–CMOD from the moment of reaching the elastic property to the ultimate deflection. There are two graph shapes defined: the first is characterized by a decrease in the destructive load with an increase in the CMOD after the appearance of the first crack (post-crack softening—pcs), and the second by an increase in strength with an increase in the CMOD (post-crack hardening—pch). A general view of a sample on the fiber composite residual strength test stand is shown in Figure 4.

### 3.2. Plate Elements

**Figure 5:**
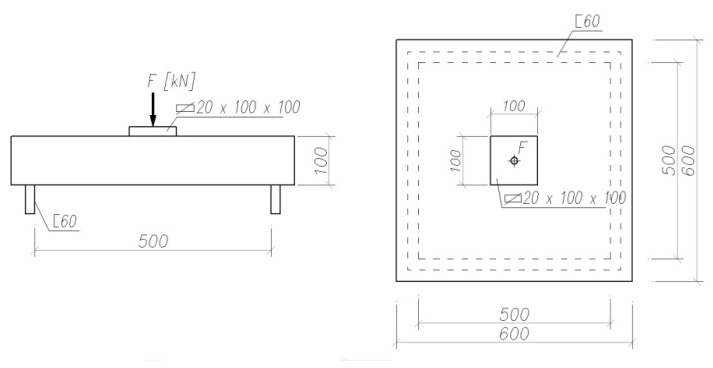
The scheme of the slab loading in the study of the energy absorption capacity of a fiber composite, recommended by [35].

The energy absorption capacity of composites with fibers is, in addition to the residual strength, an important feature determining their plasticity. This property is used in the design of fiber-reinforced concrete structures, in which, as a result of high loads, significant deformations could arise. The study of the energy absorption capacity of the fiber composite was carried out according to PN-EN 14488-5 [35]. The sample was placed in a testing machine on a rigid frame, thus obtaining a simply supported scheme around the perimeter (Figure 5). The slab was loaded with a concentrated force in the middle of its span using a rigid steel block with a thickness of 20 mm and a cross-section of 100 × 100 mm.

**Figure 6:**
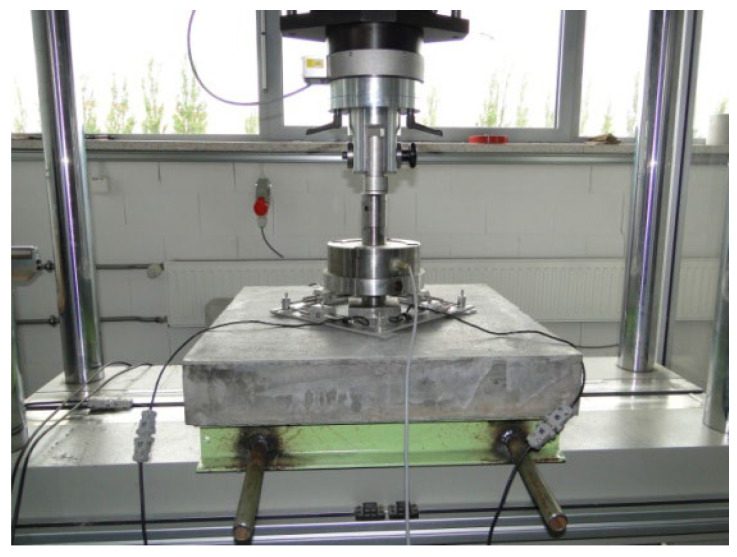
The stand for testing the energy absorption capacity of a fiber composite.

The slab was loaded continuously so that its deflection increased by 1 ± 0.1 mm/min in a controlled manner. The test ended when the slab deflection in the middle of its span reached a value of 30 mm. The increases in the load, deflection, and displacement on the upper and lower surfaces of the plate were recorded using the SAD 256 data acquisition system. The accuracy of the inductive sensors used in the tests was 1 mV/V. A general view of the stand for testing the energy absorption capacity of a fiber composite is shown in Figure 6.

## 4. Test Results and Their Analysis

Residual strength tests and their results have been widely discussed in the works [37,49,50]. The parameters of the statistical analysis of the residual strength of fine aggregate fiber composite are presented in Table 1. The obtained results clearly indicate the ductile nature of the tested material. According to the guidelines of the Model Code 2010 [31] standard, the class of the tested fiber composite can be designated as 7b (the letter “b” was determined on the basis of the f_R.3_/f_R.1_ ratio). This means that the material has a high f_R.1_ value (range 1–8) and is characterized by the “post-crack softening” feature (see Section 3.1).

**Figure 7:**
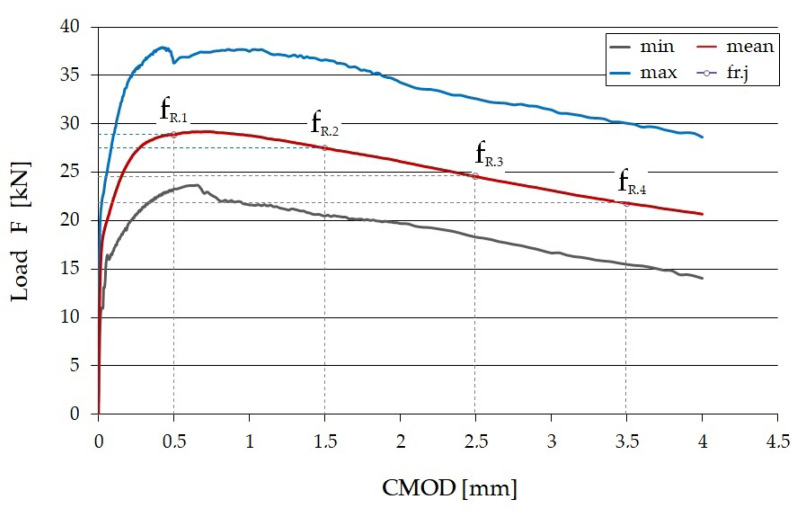
Load–CMOD relation for SFRWSC [50].

Figure 7 shows the dependence of the load (F) on the CMOD width. In order to facilitate the interpretation of the test results, the following values are shown: minimum, maximum, and average.

The shape of the chart (Figure 7) shows that for a fine-aggregate composite, a slow decrease in the destructive load was observed with an increase in the CMOD after the appearance of a crack. This confirms that it is characterized by the feature of post-crack softening. The use of fibers in the composite means that the composite does not suddenly fail, as is the case with ordinary concrete or composite without fibers.

The obtained coefficients of variation (ν), in contrast to the indices obtained in the tests of other properties of this material (from ν = 6% for the compressive strength to ν = 8% in the splitting tensile strength test), show a sufficient quality of the fiber composite used. As stated in the introduction, the fiber composites’ residual strength determined on beams have a large variability of more than 30% [61]. Such a large variability of the results of the residual strength test is a consequence of the small refraction areas formed in the beams, which was confirmed, among others, in the works [62,63].

**Figure 8:**
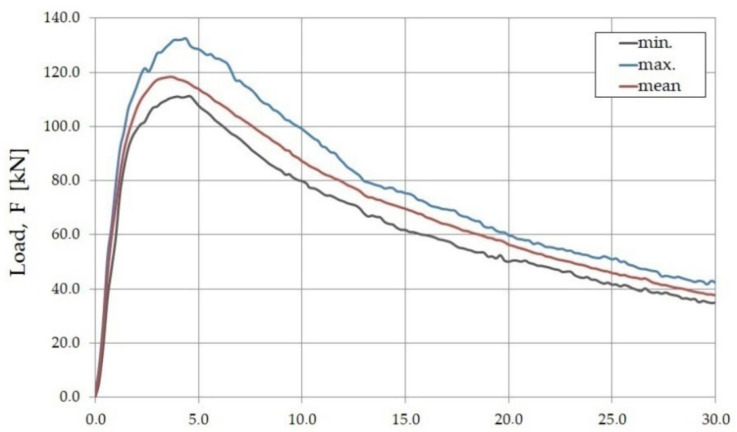
The load–deflection relation (F–δ_p.exp_) for bent slabs made from the fiber composite used in the study.

The tests of the bending plates with articulated support along the perimeter allowed for the making of a graph of the dependence of the loading force (F) and deflection (δ_p.exp_) measured in the middle of the span of the slab (Figure 8). The graph (F–δ_p.exp_) shows the mean value of all the tests, as well as the minimum and maximum values that were recorded continuously during the test every 0.5 s using the SAD 256 data acquisition system.

**Figure 9:**
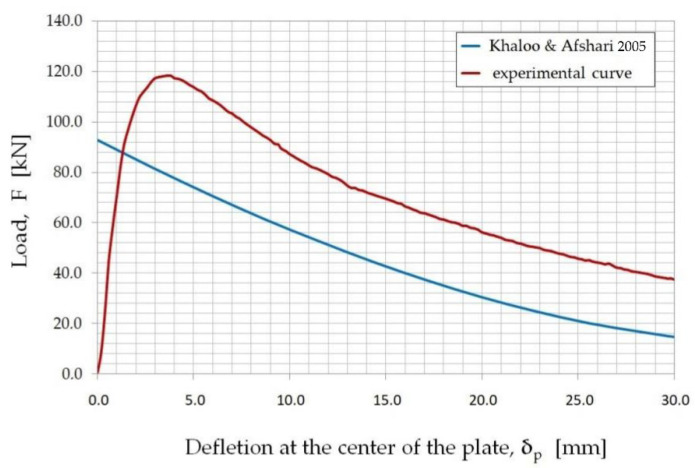
The load–deflection relation (F–δ_p,exp_) for the fiber composite covered by the research: the theoretical curve, according to Khaloo, A.R.; Afshari (2005) [64], and the experimental curve.

The relation between the loading force (F) and the plate deflection (δ_p.exp_) in the middle of the plates span was compared with the equation proposed by Khaloo and Afshari [64] describing the force–deflection relation. The graphical interpretation of the values obtained in the experiment and calculated according to [64] is shown in Figure 9.

Figure 9 shows that the tested fine-aggregate fiber composite had a greater load-carrying capacity than that determined theoretically according to the relation described in the paper [64]. The experimental load values obtained for particular deflection values were, on average, 35% higher than the theoretical values.

Based on the obtained test results, the energy absorption capacity of the plates (E_p_^exp^) of the tested fiber composite was determined. The PN-EN 14488-5 standard [35] defines this as the area under the load–deflection curve (F–δ_p_) between the deflection values (δ_p_), respectively: 0 and 25 mm. The results obtained for all plates are presented in Table 2.

The statistical analysis parameters of the energy absorption capacity (E_p_^exp^) of the fine-aggregate fiber composite are presented in Table 3. The obtained small coefficient of variation equal to 6% indicates high homogeneity of the fiber composite used in the research. The performed tests of the plates are characterized by a much smaller variation in the results (average of 9%) compared to the tests of the beams in the 3-point bending test, which was also confirmed in the works [63,64].

**Figure 10:**
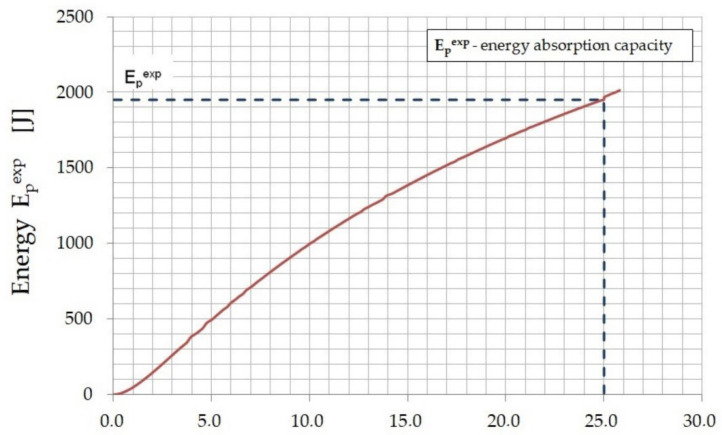
The energy (E_p_^exp^) of the deflection in the center of the plate (δ_p.exp_) for the tested fiber composite.

The energy absorbed by the fiber composite for each deflection value was determined on the basis of the mean value from the load–deflection relation diagram (see Figure 8) as the area under the curve. The relation between the energy absorbed by the plate (E_p_^exp^) and the deflection (δ_p.exp_) in the middle of the plate span is shown in Figure 10.

Such a presentation of the results allows for easy reading of the energy absorption capacity of the fiber composite (for a deflection of 25 mm) and the energy absorbed by the material for the selected deflection value, without the need to integrate the dependency graph (F–δ_p.exp_) each time.

**Figure 11:**
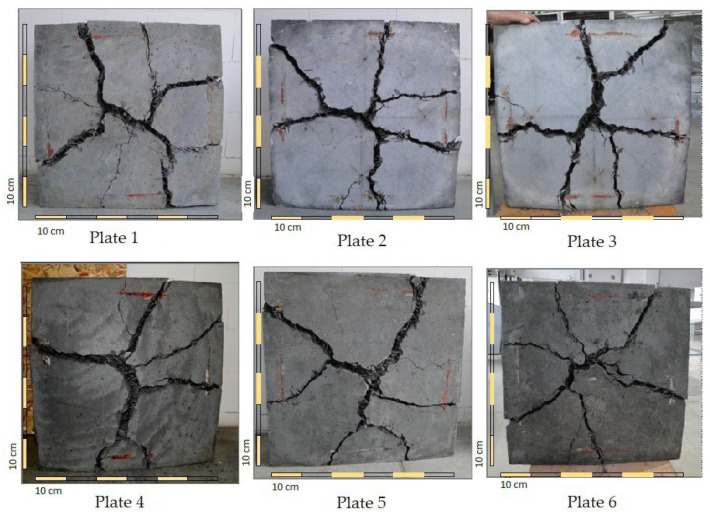
The images of the destruction of the plates made out of the fiber composite used in the study after the test of the energy absorption capacity.

Figure 11 presents images of the destruction of the bottom surfaces of the slabs after the completion of the tests. There were two to four dominant cracks in each element. This type of failure is quite typical for plates that are hinged around the perimeter and loaded evenly or with a concentrated force in the center [65].

**Figure 12:**
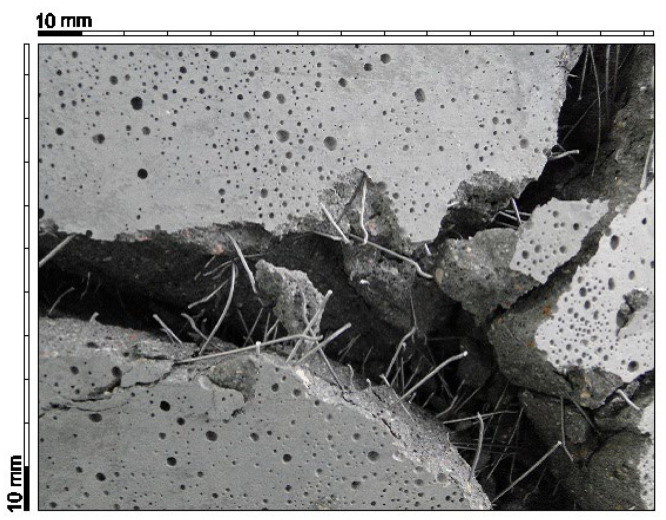
The view of the “pulled” fibers from the fiber composite matrix in a slab damaged after testing.

During the test, it was observed that as the load increased, cracks also appeared on the side and top surfaces of the slabs. The corners of the slabs went up. After the end of the test, it was observed that at significant crack widths (approximately 35–50 mm), which took place on the bottom surface of the plates, the fibers did not break but were pulled out of the matrix (Figure 12).

The PN-EN 14487-1 [66] standard enables the classification of concrete depending on the material’s energy absorption capacity, dividing it into three classes: E500, E700, and E1000. The results of the research indicate that the fiber composite covered by the experiment had a much greater energy absorption capacity (Table 3). Therefore, using the available guidelines, it is possible to assign a fine-aggregate fiber composite to the highest class: E1000. This means that the fiber composite can be used to make structural elements exposed to high static and dynamic loads. The high energy absorption capacity during the bending of the fiber composite may also reduce the dimensions of the cross-section of the designed elements.

## 5. Proposition for Determining the Residual Strengths

The methodology of testing square plates according to [35] and the method of determining the residual strength given in [30] are based on the examination of the bending elements. The graphs of the dependence of the loading force (F) and deflection (δ) obtained as a result of these tests are similar. Taking this into account, it was found that the study of the energy absorption capacity according to [35] can be used to determine the residual strengths necessary in the design of fiber composite sections.

The conducted research prompted the authors to develop a procedure for determining the residual strength based on bending square plates [67].

The plate tests allow for obtaining results with a much lower coefficient of variation than in the case of the beam test according to [30].

This procedure was based on linking the residual strengths (f_R.j_) determined for individual CMOD values with the beam deflection (δ_b.exp_) and the energy absorbed by the beam during bending (E_b_^exp^). The beam energy (E_b_^exp^) for the respective CMOD values (Table 4) was determined on the basis of the load (F)–deflection relation diagram (δ_b.exp_) as the area under the F–δ_b.exp_ curve.

The shapes and courses of the relation between the F and the δ_b.exp_ and the relation between the F–CMOD are very similar, which is confirmed by the results of the calculations presented in Table 4. They prove that the relation between the beam deflection (δ_b_) and the CMOD width, given in PN-EN 14651 [30], reflects the results of this study very well. Therefore, they can be used for further analyses.

**Figure 13:**
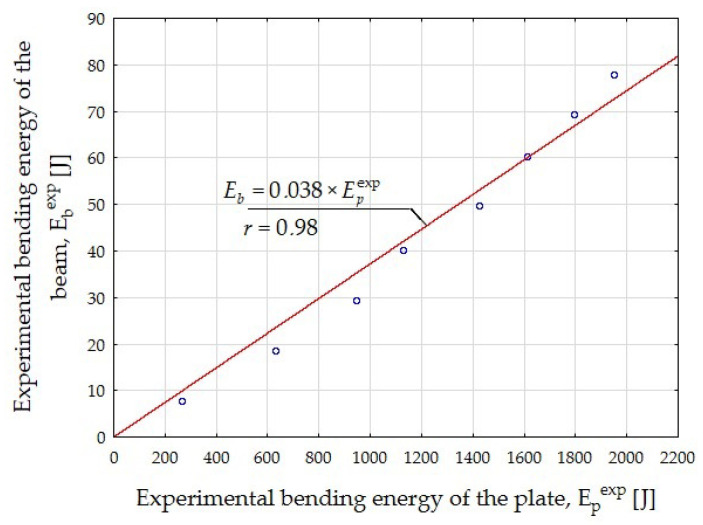
The relation between the experimental bending energy of the beam E_b_^exp^ and the experimental bending energy of the plate E_p_^exp^.

Knowing the experimental value of the energy absorbed in bending by the plate (E_p_^exp^) in relation to the deflection (Figure 10) and using the determined beam bending energies (E_b_^exp^) for the CMOD values equal to 0.5, 1.5, 2.5, and 3.5 mm, respectively (Table 4), the theoretical relation between these energies was determined (Figure 13).

The next step of the iteration was to find such plate deflection values (δ_p_) after substituting into the formula (Figure 13):

E_b_ = 0.038 × E_p_^exp^ (2)

The theoretical values of the beam bending energy (E_b_) were obtained close to the experimental values, representing the CMOD widths of 0.5, 1.5, 2.5, and 3.5 (Table 4), for which the residual strength of the f_R.j_ fiber composite was determined. It was found that the deflection values sought were δ_p_ = 3.5, 10, 15, and 21 mm, respectively.

The relation shown in Figure 13 made it possible to determine the theoretical value of the beam bending energy (E_b_) with the energy absorbed by the plate (E_p_^exp^) known from the experiment.

Knowing the theoretical values of the beam bending energy (E_b_), the mean values of the forces (F_j_) were determined for the beam deflection (δ_b_), representing the CMOD = 0.5, 1.5, 2.5, and 3.5 (Table 4) based on the physical dependence (3) of the energy stored, as shown in work [68]:

E_b_ = F_j_ × δ_b_ (3)

Next the residual strengths of fiber composite f_R.j_ were calculated according to Formula (1).

**Figure 14:**
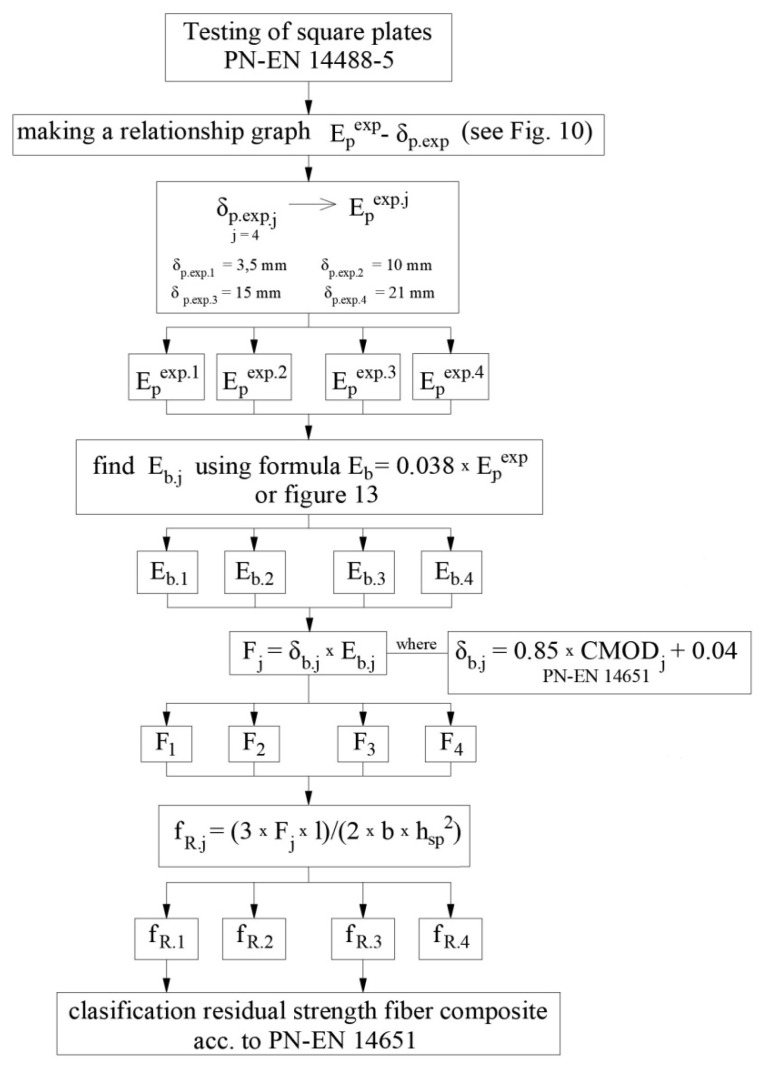
Procedure for determining the residual strength for the fiber composite.

In summary, in order to use the proposed proprietary procedure for calculating the residual strength, the flow diagram presented in Figure 14 should be considered:

The values of residual strength (f_R.j_) determined according to our own calculation procedure are presented in Table 5.

To verify the proposed procedure for testing and calculating the residual strength of a fiber composite, we used the results of the tests of the residual strengths determined on the beams for fiber composite with a steel fiber content of V_f_ = 1.2%. Additionally, we used the results of the tests of fiber composites with a steel fiber content of V_f_ = 0.5% and V_f_ = 0.9%, which were discussed in the study [49]. The results of the analyses are presented in Table 6.

The values of the residual strengths calculated in accordance with the procedure proposed by the authors are similar to those obtained in the authors’ own research and shown in the work [49]. The determined values are within the individual confidence intervals determined for the significance level α = 0.05. It can therefore be concluded that the differences between the theoretical and experimental values are statistically insignificant. Moreover, the obtained coefficients of variation (ν) are much smaller (1–8%) than those obtained in the beam tests (10–17%) (Table 1), which indicates a much smaller dispersion of the residual strength results.

## 6. Conclusions

The results of the study of the centrally bending square slabs simply supported at the perimeter show that the slabs were characterized by a much lower coefficient of variation compared to the results of the beam tests according to PN-EN 14651. This difference was due to the fact that in the tests of fiber concrete in the extra-elastic range, in the case of beams on a small fracture area, only a small number of fibers were active during the destruction. This phenomenon occurred to a much lesser extent in the case of the plates or full-size elements.

Taking into account that the methodology of testing plates and beam sand the shapes of the loading force–deflection relation (F–δ) are similar, it can be assumed that the test energy absorption capacity can also be used to determine the residual strength, characterized by a much lower coefficient of variation than in the case of beams.

The shapes of the F–δ diagrams obtained in the authors’ own research indicate that after cracking, the fine-aggregate fiber composite had a slow decrease in the destructive force with increasing deflection. This was also noticed during the test of the residual strength on the beams. The slight decrease in the curve after obtaining the maximum value of the load is related to the number of fibers in the fiber composite.

The essence of the author’s proposed method of determining residual strength for the fiber-reinforced cement composite consists of relating the energy absorbed by the beam when bending with a deflection corresponding to the width of the CMOD crack, for which the residual strengths are determined with the energy absorbed by bending panels at equivalent deflections. Then, on the basis of the determined absorption energy in the beam, the value of the load corresponding to the successive energies is determined, and as a last resort—the residual strength.

The novel proposition for determining the residual strength with the use of centrally bending square plates simply supported at the perimeter presented in the article is an alternative to the 3-point bending of the beam using the method specified in PN-EN 14651. The proposed test method is aimed at a more realistic modeling of biaxial bending than in the case of beams. In this study, the obtained residual strengths are characterized by coefficients of variation not exceeding 8%, while according to PN-EN 1465, they reach 17%.

The authors are aware, however, that the presented method of determining the residual strength, based on the energy absorption capacity of the fiber composite, is not perfect. Further experimental and analytical works are planned to improve the method, inter alia, by introducing appropriate factors to facilitate the described procedure. The great advantage of this experimental–analytical procedure is that the residual strengths defined in this way are characterized by a low coefficient of variation.

## Figures and Tables

**Table 1:** Results of the flexural tests.

Statistical Parameters	f_R.1_	f_R.2_	f_R.3_	f_R.4_	f_LOP_
	CMOD (mm)				
	0.5	1.5	2.5	3.5	
Mean value (MPa)	9.27	8.80	7.87	6.98	6.34
Standard deviation: s (MPa)	1.20	1.29	1.25	1.16	0.67
Coefficient of Variation: v (%)	13	15	15	17	11
Minimal value (MPa)	7.30	6.68	5.82	5.07	5.24
Confidence interval (MPa)	8.82 ÷ 9.74	8.30 ÷ 9.28	7.39 ÷ 8.34	6.53 ÷ 7.42	6.09 ÷ 6.60
f_R.3_/f_R.1_	0.79				
f_R.1_/f_LOP_	1.39				
Classification of fiber composite according to fib Model Code 2010 [30]: **7b**					

**Table 2:** Test results for square plates made of fiber composite.

Specimen	Maximum Load: F_max_ (kN)	Deflection δ for F_max_ (mm)	Energy E for F_max_ (J)	Energy Absorption Capacity E_p_^exp^ (J)
P1	111.1	4.4	450	2084
P2	121.4	3.0	280	1822
P3	116.2	3.6	330	1924
P4	132.6	4.4	470	2102
P5	119.4	3.4	310	1818
P6	116.3	3.8	340	1923
Mean value	**119.5**	**3.77**	**360**	**1945**

**Table 3:** Statistical parameters of the energy absorption capacity E_p_^exp^ of the fiber composite used in the study.

Statistical Parameters	Energy Absorption Capacity E_p_^exp^
Mean value (J)	1945
Standard deviation, s (J)	113
Coefficient of variation, v (%)	6
Minimal value (J)	1818
Confidence interval (J)	1815 ÷ 2075

**Table 4:** The CMOD and the corresponding beam deflection values (δ_b_), (δ_b.exp_) and bending energy (E_b_^exp^).

CMOD (mm)	Deflection (mm)	Experimental Deflection (mm)	Energy Absorption Capacity (J)
	δ_b_ = 0.85 · CMOD + 0.04 [30]	δ_b.exp_	E_b_^exp^
0.5	0.46	0.48	11.3
1.5	1.31	1.34	35.6
2.5	2.16	2.17	58.3
3.5	3.00	2.99	72.4

**Table 5:** The residual flexural tensile strengths (f_R.j_) of the fiber composite calculated on the basis of the proposed procedure.

1	2	3		4	5	6
Deflection δ_p_ (mm)	Plate Bending Energy (J)	Beam Bending Energy (J)		Beam Deflection δ_b_ (mm)/CMOD (mm)	Load F_j_ (kN)	Residual Flexural Tensile Strengths (MPa)
	E_p_^exp^	E_b_	E_b_^exp^			
3.5	343.6	13.1	11.3	0.46/0.5	27.8	f_R.1_ = 8.89
10	977.4	37.1	35.6	1.31/1.5	28.1	f_R.2_ = 9.00
15	1384.4	52.6	58.3	2.16/2.5	24.2	f_R.3_ = 7.76
21	1762.8	67.5	72.4	3.02/3.5	22.4	f_R.4_ = 7.16

E_b_—value determined on the basis of the author’s dependence: E_b_ = 0.038 · E_p_^exp^ (see Figure 13). E_p_^exp^—energy value obtained from the experiment (Figure 10) for the appropriate CMOD values (see Table 4).

**Table 6:** Experimental and calculated values of residual flexural tensile strengths of the fiber composite.

V_f_(%)	Residual Flexural Tensile Strength (MPa)	Values Obtained in Own Research and According to [49]				Values Obtained on the Basis of Calculations According to Own Procedure		
		Mean Value (MPa)	Standard Deviation: s (MPa)	Coefficient of Variation: v (%)	Confidence Interval (MPa)	Mean Value (MPa)	Standard Deviation: s (MPa)	Coefficient of Variation: v (%)
1.2	f_R.1_	9.27	1.2	13	8.82 ÷ 9.74	8.89	0.5	5
	f_R.2_	8.80	1.29	15	8.30 ÷ 9.28	9.00	0.1	1
	f_R.3_	7.87	1.25	15	7.39 ÷ 8.34	7.76	0.4	5
	f_R.4_	6.98	1.16	17	6.53 ÷ 7.42	7.16	0.5	6
0.9 [49]	f_R.1_	7.08	1.2	16	6.16 ÷ 8.00	7.11	0.5	8
	f_R.2_	6.96	0.94	13	6.24 ÷ 7.68	7.68	0.1	1
	f_R.3_	6.37	0.95	14	5.65 ÷ 7.10	6.70	0.2	3
	f_R.4_	5.72	0.86	14	5.06 ÷ 6.37	6.17	0.2	4
0.5 [49]	f_R.1_	4.51	0.56	12	4.07 ÷ 4.96	4.64	0.3	7
	f_R.2_	4.72	0.58	12	4.26 ÷ 5.17	5.15	0.2	4
	f_R.3_	4.61	0.48	10	4.24 ÷ 4.99	4.67	0.2	5
	f_R.4_	4.38	0.50	11	3.99 ÷ 4.77	4.29	0.3	6

## Data Availability

The data presented in this study are available on request from the corresponding author.

## Nomenclature

CMOD	crack mouth opening displacement
SFRWSC	steel fiber-reinforced waste sand concrete
*F*	load
*E*	bending energy
*E_p_*	theoretical bending energy of the plate
*E_b_*	theoretical bending energy of the beam
*E_p_^exp^*	experimental bending energy of the plate
*E_b_^exp^*	experimental bending energy of the beam
*f_R._*_1_ *f_R._*_2_ *f_R._*_3_ *f_R._*_4_	residual strength determined in accordance with relevant standard for CMOD = 0.5 mm, 1.5 mm, 3.5 mm, and 3.5 mm
*h_sp_*	distance between the tip of the notch and the test specimen in the mid-span section
*l*	length of span
*b*	width of the beam
δ	deflection
δ_b_	theoretical deflection at the center of the beam
δ_p_	theoretical deflection at the center of the plate
δ_b.exp_	experimental deflection at the center of the beam
δ_p.exp_	experimental deflection at the center of the plate
*s*	standard deviation
ν	coefficient of variation
